# Purification of Lipid Rafts from Cultured Cells

**DOI:** 10.1100/tsw.2002.846

**Published:** 2002-06-18

**Authors:** John Graham

**Affiliations:** School of Biomolecular Sciences, Liverpool John Moores University, UK

**Keywords:** lipid rafts, membrane vesicles, plasma membrane, cultured cells, detergent-resistant membranes, Triton X-100, OptiPrep^TM^, iodixanol, discontinuous gradient

## Abstract

Lipid-rich lipid rafts are microdomains of the plasma membrane that are resistant to low concentrations of nonionic detergent. This forms the basis for their isolation. Either a microsomal fraction or a postnuclear supernatant are loaded beneath a discontinuous iodixanol gradient. If all the solutions contain 0.5–1.0% Triton X-100, the intact lipid rafts float to the top of the gradient while all of the other detergent-solubilized membranes remain at the bottom.

**METHOD TYPE:** extraction, isolation, purification and separation

**SUB METHOD TYPE:** centrifugation

## INTRODUCTION

The importance of lipid-rich microdomains of the plasma membrane in signal-transduction events, in lipid transport and in various internalization processes and in the regulation of plasma membrane-cytoskeleton interactions have only become well established over the last 5 years. A number of important cholesterol and sphingolipid-rich structures have been identified and studied, notably caveolae and lipid rafts. The isolation of caveolae using OptiPrep™ is described in Ref. [1].

New methods have been recently developed for the isolation of these lipid rafts based on the insolubility of these structures in the nonionic detergent TritonX-100. Either the intact cells are treated with a detergent-containing solution or a postnuclear supernatant is prepared from a cell homogenate and then Triton X-100 is added to this supernatant. The former approach was adopted by Oliferenko et al.[2] for EpH4 cells (a spontaneously immortalized mouse mammary epithelial cell line); the latter by Lafont et al.[3] for fowl-plague infected MDCK cells. The detergent-treated material is then adjusted to a high density and layered under a discontinuous iodixanol gradient. The lipid rafts, which have a relatively low density, float away from soluble proteins and detergent-insoluble cytoskeleton-associated proteins, which remain in the load zone.

Oliferenko et al.[2] used the technique to study the association of hyaluronic acid cell surface receptor CD44 and annexin II with lipid rafts and their interaction with the cytoskeleton. Lafont et al.[3] investigated the role of lipid rafts in apical trafficking and in particular their association with SNAP receptors. The following protocol is based on both of these published methods.

## MATERIALS AND EQUIPMENT

OptiPrep™

Isolation Medium (IM): 150 mM NaCl, 5 mM dithiothreitol (DTT), 5 mM EDTA, 25 mM Tris-HCl, pH 7.4 supplemented with a cocktail of protease inhibitors (see Note 1)

Triton X-100

Phosphate-buffered saline (PBS)

Cell homogenizer: Cracker (ball-bearing homogenizer), or syringe with fine needle, or tight-fitting Dounce homogenizer (see Note 2)

Ultracentrifuge with any small volume (approx. 4 ml) swinging bucket rotor (e.g., Beckman SW60Ti, Sorvall TH660 or equivalent)

Syringe with metal cannula (for underlayering) or plastic Pasteur pipette (for overlayering)

## METHOD

Carry out all operations at 0–4°C.

### Isolation from a Total Cell Lysate

1. Adjust IM to 1% Triton X-100.
2. Wash the cell monolayer twice with PBS and scrape into this medium.
3. Pellet the cells and resuspend in 0.2 ml of IM + Triton; then leave on ice for 30 min.

### Isolation from a Postnuclear Supernatant

1. Homogenize the cells in IM (see Note 2).
2. Centrifuge the homogenate at 1000g for 10 min.
3. Adjust the supernatant to 1% Triton X-100 and leave on ice for 30 min.

### Density Gradient

1. Add 4 vol of OptiPrep™ to 2 vol of either the homogenate or 1000*g* supernatant.
2. Dilute OptiPrep™ with IM + Triton to give 35, 30, 25, and 20% (w/v) iodixanol (see Note 3).

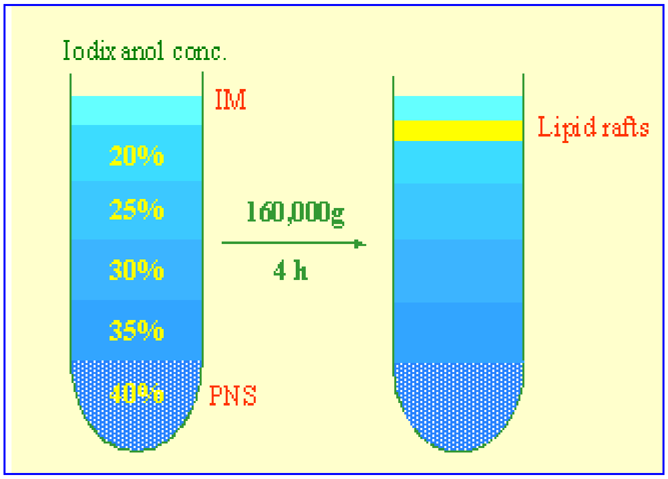

**FIGURE 1**. Isolation of lipid rafts by flotation from a postnuclear supernatant (PNS) in a discontinuous iodixanol gradient. IM = Isolation Medium. See text for more details.

3. In tubes for the swinging-bucket rotor layer 0.6 ml each of the sample, the four gradient solutions and IM + Triton to fill the tube.
4. Centrifuge at 160,000$g_{av}$ for 4 h (see Note 4).
5. Collect the lipid rafts from the top interface (see Fig. 1).

## NOTES

1. The isolation media used by both Oliferenko et al.[2] and Lafont et al.[3] were similar, although the level of DTT used by Oliferenko et al. was 1 mM rather than 5 mM and EDTA was omitted. Protease inhibitors such as PMSF, leupeptin, antipain, aprotinin, etc. should be included in all of the media.
2. Use a small Dounce homogenizer or passage through a fine gauge syringe needle or a cell cracker to effect homogenization. For more information on the homogenization of cultured cells see Ref. [3].
3. The gradient in the protocol is as described by Oliferenko et al.[2]. Lafont et al.[3] used slightly fewer steps of either 30% iodixanol and IM + Triton or 30, 20, and 5% iodixanol. In all cases the lipid rafts band close to the top of the gradient. Some variations on this gradient strategy, and the cell types to which they have been applied, are given in Table 1. Whether a multiple step gradient is required, or whether a simpler two-step system (Table 1) might suffice depends on the operator's requirements. Because it is now recognized that there are different types of low-density domain the original method by Oliferenko et al.[2] may be more appropriate. In a new modification by Lindwasser and Resh[13] the sample was adjusted to 50% iodixanol and overlayered with 40, 30, 20, and 10% iodixanol. By harvesting the gradient in small volume fractions subfractions of these lipid-rich domains that displayed a heterogeneous cholesterol, GM1 glycolipid and caveolin-1 content, were identified.
4. Oliferenko et al.[2] used a longer centrifugation time of 12 h at a slightly lower RCF (120,000$g_{av}$). Because of the relatively short sediment path length of the rotor, 4 h at the higher RCF is probably satisfactory, but this time requirement may vary with the mode of preparation and cell type. Some variations on these centrifugation conditions, and the cell types to which they have been applied, are given in Table 1.

**TABLE 1**
**Selected Publications on the Isolation of Lipid Rafts in OptiPrep™ Gradients**

| Iodixanol gradient | Centrifugation | Source material | Ref # |
|---|---|---|---|
| 20%,25%,30%,35% | 160,000g/4 h | BHK cells | 4 |
| 20%,25%,30%,35%,40% | 250,000g/2.5 h | MDCK cells | 5 |
| 24%,21%,15%,6% | 150,000g/5 h | Drosophila | 6 |
| 30%,35% | 170,000g/4 h | Human breast carcinoma cells | 7 |
| 30%,40% | 250,000g/2 h | Yeast | 8 |
| 30%,40% | 250,000g/2 h | Oligodendrocytes | 9 |
| 30%,40% | 250000g/2 h | Rat basophilic leukemia cells | 10 |
| 30%,35% | 170,000g/4 h | COS-1, Jurkat cells | 11 |
| 30%,35% | 140,000g/20 h | Human embryonic kidney cells | 12 |

## ACKNOWLEDGEMENTS

The author and TheScientificWorld wish to thank Dr. Lukas Huber, IMP, Research Institute of Molecular Pathology, A-1030, Vienna, Austria and Dr. Kai Simons EMBL, Cell Biology and Biophysics Program, 69117 Heidelberg, Germany for their kind cooperation in the preparation of this Protocol Article and Axis-Shield PoC, AS, Oslo, Norway for their kind permission to adapt OptiPrep™ Application Sheet S20 and Table 38 from the Axis-Shield Catalogue 2002.
